# Neural network modeling of psychoanalytic concepts

**DOI:** 10.3389/fnsys.2025.1585619

**Published:** 2025-07-24

**Authors:** Daniel S. Levine, Ana Maria C. Aleksandrowicz, Ana Luiza S. Verissimo Lopes

**Affiliations:** ^1^Department of Psychology, University of Texas at Arlington, TX, United States; ^2^FIOCRUZ, Rio de Janeiro, Brazil; ^3^UNIRIO, Rio de Janeiro, Brazil

**Keywords:** neural networks, psychoanalysis, brain, ego, prefrontal cortex, amygdala, hypothalamus

## Abstract

Techniques used over decades in brain-based neural network modeling are applied to understanding processes involved in psychoanalysis. Behavioral change is interpreted as a transition, using simulated annealing, from a less to a more optimal attractor in a competitive-cooperative dynamical system that includes analogs of the amygdala, prefrontal cortex, and hypothalamus, and the neurotransmitter norepinephrine. The article explores how psychoanalysis can facilitate the quest for the life that is as meaningful as possible. The resulting network theory allows for new understanding of several traditional Freudian concepts. The theory provides insights about the life and death drives. It also helps us understand object and narcissistic libido, and the contrast of healthy forms of libido based on autonomy vs. unhealthy forms based on dependence. This inquiry relates to the balance between self-interest and empathy, mediated by various areas of the limbic system. It illuminates transference, which involves both an emotional and intellectual relationship between the analyst and analysand, mediated by cognitive-emotional interactions in amygdala and orbitofrontal cortex. Sublimation, or redirection of socially inappropriate urges toward more appropriate behaviors, is interpreted via lateral inhibition between representations of similar complex behaviors.

## Introduction

It is a challenging and fascinating intellectual mission to try to approach psychoanalytic concepts with more scientific ones. Psychoanalysis insists on its double essential nature, as a science and an art; it is also deeply embedded in a philosophical approach to how to experience a meaningful life. Its main aim is to provide the individual with psychological autonomy, the indispensable precondition for a person to be truly a compassionate human being, really caring for the other, in society. But, every individual shares a common nervous system with all of the human species (even if the nervous system, naturally, works in different ways in each individual, it always follows common rules). As a science, the principles which guide human brain functioning are universal, so psychoanalysis can and must dialogue with scientific models. In this sense, we can speak of “modeling” psychoanalytic concepts.

Before he developed the psychoanalytic framework, Freud in his *Project for a Scientific Psychology* (Freud, 1895) sought to base the understanding of the human mind on neurology and functions of the brain. As Pribram and Gill (1976) demonstrate, this work was remarkably harmonious with the state of the art in neuroscience at the time. Yet this was a time before the firm establishment of current knowledge about electrical transmission in neurons and plasticity of synapses, let alone detailed functions of specific brain regions such as prefrontal cortex and amygdala. Hence it is our goal to bring to bear more recent knowledge of systems neuroscience on the understanding of several ideas from classical Freudian psychoanalysis.

It is important to highlight that the psychoanalytic framework has been interpreted and reinterpreted over time. However, Freud established, on more than one occasion, the theoretical pillars that characterize psychoanalysis. He established that the “essence” of these “pillars” must be kept in each new reinterpretation of psychoanalytic theory and practice. Of course, inevitably, the concepts that compose those pillars suffered variations of meaning according to the evolution of the comprehension of the terms over time and even inside a certain framework of psychoanalytically oriented ideas that can be diversely interconnected. Freud himself changed his own definitions of various psychoanalytic concepts and psychic dynamics over the more than 30 years in which he had been improving his oeuvre.

Each new post-Freudian psychoanalytic school conducted controversial changes—without ever denying their essential importance—to the meaning and interconnections of the concepts that constitute the psychoanalytical pillars. Frequently, they chose their Freudian references, with vast theoretical freedom. In this paper, all the psychoanalytical concepts presented result from an original interpretation of Freud’s oeuvre, preserving the “essence” of the Freudian ideas. We have recognized and honored our debt with a Freudian legacy—and we have used the complete theoretical and conceptual Freudian framework. However, we admit that our interpretation dwells within a thin frontier between conceptions better accepted as Freudian ones and those more assumed as heterodox innovations. Because of that, we named ourselves Freud’s Forwarders—not followers. Mainly, what we want the most is to share with people interested on our re-reading of Freud’s oeuvre its possible benefits in terms of increasing our psychic energy—our Life Trieb—which we need to have the vital strength (the joy, the hope, the guts, the enthusiasm that turns deceptions into challenges) to continue trying to accomplish projects that can give meaning to our lives as mature individuals and as members of the human kind. For the current article, we revisited mainly the following of Freud’s works: *Project for a Scientific Psychology* (Freud, 1895); *Three Essays on the Theory of Sexuality (* Freud, 1905*); Five Lectures on Psycho-Analysis* (Freud, 1910); *On Narcissism: an Introduction* (Freud, 1914); *The Unconscious* (Freud, 1915); *Beyond the Pleasure Principle* (Freud, 1920); *The Ego and the Id* (Freud, 1923).

We must also emphasize that the psychoanalytic model that is the basis to our discussion in this paper is founded in Spinoza’s philosophy (Kisner, 2018; Spinoza and Hampshire, 1996), according to which all that exists in the universe is composed by only one Substance that Spinoza’s calls nature. There are infinite “attributes” that allow different beings in the Universe to recognize the reality. Human beings have access to two attributes: Extension (or “body”) and Thought (or “mind”). But, these are only two human ways—through what can be called our “mind” and “brain”, for instance—of the same substance.

So, in epistemological terms, when we speak in “mind” and “body”, we are speaking of a total correspondence between two aspects of reality that we can study and deal with through different methods and resources, but there is, in principle, an absolute correspondence between them.

In this sense, certainly we can consider Psychoanalysis as a theory of the mind, and of interactions between mental constructs and concepts. There is considerable debate among scientists, philosophers, and spiritual leaders about the relationship between the mind and the body, and more specifically between the mind and the brain. Yet there is general agreement that there are characteristic brain processes and interactions that correlate with mental events. In this article, as stated above, we fully endorse this understanding. The growth of physiological techniques over the last five decades, including magnetic resonance imaging of brain regions while people are performing specific cognitive and behavioral tasks, has vastly increased our knowledge of the roles played by specific brain regions in our thoughts and emotions.

The last five decades have also seen a growth of computational and mathematical theories of mind-brain interactions by means of neural networks (Levine, 2019). Neural networks are widely known as a set of techniques for mimicking biological systems to use in engineering and artificial intelligence applications. Yet these networks are equally important for understanding natural intelligence, including both thoughts and feelings and their interactions.

Over the last half century, there has been progress in computational modeling of cognitive and behavioral functions such as perception, conditioning, memory, and decision making (Levine, 2019). Models of these phenomena started out as abstract cognitive constructs, but as relevant neuroscientific data emerged, gradually incorporated what was known about the psychological functions of specific interacting brain regions.

Neural network models of psychological functions are typically based on dynamical systems of equations describing interactions between relevant neural or psychological variables, causing those variables to change over time. These variables can either denote the strength of activations of particular brain regions or the intensity of psychological constructs such as memories or emotions. The interactions between these variables are non-linear, meaning that a small change in one variable can in principle produce larger changes in an interacting variable (Galatzer-Levy, 2017).

Over time, typical dynamical systems sometimes reach a stable equilibrium state, where the numerical intensities of the variables reach a constant level and stay there. Sometimes instead the systems tend toward a periodic solution, wherein the variables oscillate between states. Or they exhibit chaotic behavior, in which they do not settle into any stable pattern.

In our psychoanalysis model, there are two main libido dynamics that describe our affective life. These are the struggles (that can be sometimes creative and at other times prejudicial to a full life) between the energies directed at enhancing life and those directed toward breakdown, which we call the Life and Death Triebe,^1^,^2^ and between the more positive or negative forms of narcissistic libido (responsible for self-esteem and/or selfish ego in variable proportions) and object libido (responsible for heteronomous and/or dependent relationships). We must remember all these struggles take place in an unconscious level (that can be shared with the conscious one, especially if the individual exercises this internal dialogue, through psychoanalysis or other form of contact with his/her unconscious, as art or mysticism).

For psychoanalysis, the equilibrium states, also known as attractors, are of particular interest because they represent how the unconscious deals with these two interconnected libido dynamics, what will be reflected in his/her behavior patterns or feelings (Galatzer-Levy, 2017). The aim of psychoanalytic treatment is often to change the unconscious processes (what will be, usually, expressed through behavioral or mental patterns) from one attractor to another—presumably, to another attractor that is better integrated and serves the person’s goals in a more positive manner.

Attractors could refer to the state of a person’s whole mind or to a pattern of behavior or thinking regarding some aspect of the mind (for example, attitudes toward one’s parents or one’s work). Thus, for example, the Life and Death Triebe could be thought of as competing attractors for the large system of the mind. Yet there are also more detailed attractors denoting patterns for dealing with work, family, or romantic relationships, for example. There may be more than two attractors for decisions about any particular aspect of life, attractors which have various relationships with the life and death impulses. Hence, the study of complex dynamical systems argues against the simple dichotomy of Life and Death Triebe. The relationship between these two fundamental Triebe—Life and Death—is extremely important to psychoanalysis and is the subject of deep research but, without any doubt, we cannot reduce it to any simplified dichotomized processes.

### Moving between attractors

**FIGURE 1:**
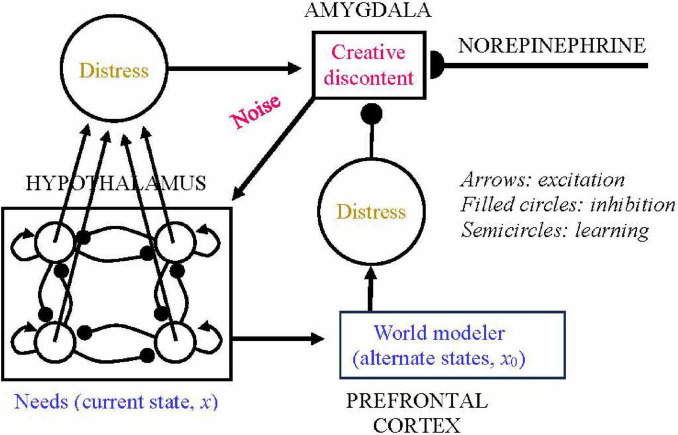
Tentative neural network for self-actualization (modified from Levine, 1994).

Levine (1994, 2005, 2009) developed a neural network model of the process of changing attractors (Figure 1). The network dynamics through time are based on interacting representations of the organism’s basic needs; of current and imagined mental states; of emotional reaction to those current and imagined states; and of the motivation or initiative toward changing mental state. All of these representations can be tentatively identified with specific brain regions, and the quantities in the model represent activations of those areas. At the current state of knowledge, the quantities in this model are not assumed to be specific measurable biological variables. Also, this model has not been simulated by actual computer runs of the underlying equations. But if our model is correct, it can guide the way toward more precise biological models as more knowledge becomes available.

The square in the lower left of Figure 1 shows an idealized representation of the various needs of a person. These include physiological needs such as food, sex, and safety as well as psychological needs such as love, esteem, belonging, and self-actualization: the idea bears some resemblance to the hierarchy of needs due to Maslow (1968). These needs are assumed to be located in the hypothalamus and brain stem, which have strong connections with other internal organs (heart, digestive system, endocrine glands, and skin) and contain nuclei that are important for basic drives (Thompson, 2000, Chap. 7).

The mental state of a person at a given time is modeled as a set of quantities mapping the activation of the different needs. To simulate the intuitive notion that some mental states are more satisfying than others, we posit a mathematical function that we call V corresponding to a kind of “distress” level from unmet needs of the organism. The aim of psychoanalysis or any other treatment, then, is to move the network representing the organism’s needs from an attractor with a higher value of V to another attractor with a lower value of V. This movement of the network corresponds to replacing a maladaptive behavior pattern with one that is more suited to the organism’s goals.

The attractor framework applies to any dynamical system describing any types of variables. They could be populations of animals, locations of subatomic particles, or amounts of either happiness or suffering. The attracting states have to do with the person’s overall adjustment. So the framework does not exclude any type of variable in the individual.

We must remark that the term “adjustment” has to be understood in a very dynamical sense in psychoanalysis, because the ultimate goal in a psychoanalytic process (not “treatment”) is not social or even personal “adjustment”, but the emotional growth of an individual to reach his/her own condition of affective autonomy in order to really contribute to the his/her and the others’ happiness. It cannot be reached without taking risks and even, perhaps, suffering from some unpleasant consequences of abandoning from his/her previous behavior patterns until he/she can find another equilibrium condition.

There are many neural network models that include a mathematical function such as V, which is often called an energy function by analogy with physics or a Lyapunov function after the Russian mathematician who was one of the originators of the concept (Cohen and Grossberg, 1983; Hopfield and Tank, 1985; Rumelhart et al., 1986). A Lyapunov function is a function of the network variables that decreases over time due to the equations defining the dynamics of the variables. Ultimately, the system goes close to one of the attractor states and stays there over time. But it is not guaranteed to go to the “optimal” attractor, that is, the attractor with the minimum value of the distress level V.

If the dynamical system described by the needs module goes to a non-optimal attractor over time, it will not move toward the more optimal state corresponding to more adaptive behavior without some outside forces attracting on it, forces represented by other parts of the network of Figure 1. Some neural networks use such an outside force involving random noise, via a mathematical schema called simulated annealing (Hinton and Sejnowski, 1986).

Levine (1994, 2005, 2009) developed and interpreted biologically a continuous simulated annealing scheme for moving from a less to a more optimal attractor (Figure 1). For this move to occur, the network or organism needs to be aware of another state of the needs module that is more adaptive, that is, has a lower V function, than the current state of that module. Imagined alternative states are assumed to reside in a “world modeler” module somewhere in the prefrontal cortex, perhaps in its dorsolateral area (DLPFC; Fuster, 2000). The world modeler imagines and makes “copies” of various possible states of the need subsystem and calculates the Lyapunov function for each, in search of a state with a lower V (i.e., a state that is closer to optimal). If V of the current state, call that state x, is larger than V of some other projected state, call it x_0_, the combination of excitation from the needs module and inhibition from the world modeler produces a signal of magnitude V(x)-V(x_0_) to another module that represents a desire for change. We call the latter module “creative discontent” and identify it with a part of the amygdala, because that brain region represents positive or negative emotional valences of stimuli or actions (Gaffan and Murray, 1990).

When thus activated, the discontent module in turn sends random noise back to the needs module, which can move that module out of a suboptimal local minimum. Yet the noise may or may not be sufficient to cause a move to a global minimum or any other attractor. Whether that move occurs depends on both the dynamics of the needs module and the intensity of signals from creative discontent activity to the noise signal generator.

The intensity of the noise signals in turn depends on two other variables that influence the amygdala. One of these variables is the strength of signals to the amygdala from the prefrontal cortex, the brain’s seat of organization and planning. Clinical observation of prefrontally damaged patients shows that they often express frustration when their actions are ineffective, but this frustration does not lead them to change their actions (Milner, 1964).

The other variable that influences the strength of simulated annealing noise is the strength of signals from the *locus coeruleus*, a region of the midbrain that sends out the chemical neural transmitter norepinephrine (NE). NE is the transmitter most associated with non-specific arousal and with exploration (see, e.g., Aston-Jones and Cohen, 2005), and therefore we identify it in our mathematical theory with a variable called “initiative” that simulates the strength of the organism’s motivation to change attractors. In the network of Figure 1, the needs module is competitive, that is, each need representation tends to inhibit the others. Based on some mathematical theorems about competitive neural networks (Grossberg, 1973), dynamics of the needs module are likely to be more winner-take-all (i.e., only one or a few nodes have non-zero activity over time) with a low NE level, and more coexistent (i.e., many nodes have nearly equal activities over time) with a high NE level.

In the psychoanalytic context, this means that a larger NE level, up to some point that leads to stress, moves the module toward attractors that satisfy a greater number of needs. These would be attracting states that move the individual away from mere survival toward richness of life and fulfilling interpersonal relationships. Adding this richness inevitably involves facing risks.

### The search for meaning in life

Freudian Psychoanalysis is a theory that is usually understood in very limited terms. It is common for authors to consider Freud’s Life Trieb, or Eros, just as a drive associated with the sexual instinct, as well as instincts to engage in other pleasurable activities. By contrast, the life Trieb also drives creative impulses. His Death Trieb, or Thanatos, is usually understood as particularly manifested in aggression, apathy, and/or self-destructive behavior. This aggression is normally directed outward to other people, but also can be directed inward in the form of suicide or depression. But these are very basic definitions of the extension of meanings that Life and Death Triebe can present. Freud’s conception of the Life and Death Triebe, as well other capital Freudian concepts, evolved during his lifetime and is still evolving in the work of modern neo-Freudian interpreters, including our own work (Aleksandrowicz and Levine, 2005, 2012; Aleksandrowicz and Bellinello, 2011).

One of our long-term goals is to understand what properties of the brain’s neural network correspond to a feeling that life is meaningful. This is an important question for psychoanalytic practice, as some of us treat young analysands who have considered suicide because of a feeling that their life is meaningless. Meaningfulness corresponds to “a state that will meet most of our needs: the needs for excitement, delight, and adventure as well as the needs for emotional security, food, shelter, and love” (Levine, 2018, p. 123). But to psychoanalysis this means also the capacity of facing the Death Trieb in such a way that can turn it in favor of Life Trieb. This is such a daring inner challenge that must be considered one of the greatest quests of contemporary psychoanalysis.

Ultimately, we hope to describe this meaningful state in terms of mathematical dynamical systems. Such a description is elusive because the underlying system is incredibly complex, but we can get some intuition for what it would entail based on what is desirable in human mental life. A meaningful state includes a stable equilibrium in some dimensions of life, but also an especial skill in taking positive advantages of facing apparently unstable, challenging mental states Steadiness in ethical values (and living by those values) is desirable, for example, but there are situations where it is needed to go beyond traditional values to create a new set of ethical references. So are stable creative work interests and stable personal relationships—Freud’s love and work (*lieben und arbeiten*). Yet being stuck in a completely unchanging mental state corresponds to stagnation, which is undesirable. Instead, the meaningful life is open to new experiences and the possibility of change in other dimensions, always facing some inner risks, what is an essential dimension to psychoanalysis. The person living meaningfully is always facing and creating new internal psychodynamics, which can be accomplished by meeting new people, going to new places, and reading new books, some of which become significantly ingrained in the synapses of their brains. But we cannot confuse the mere external behavior with an internal psychodynamic: an active social life, for instance, if the same pattern of internal dynamics is repeated from group to group, changes relatively little in making a life really meaningful (but, of course, in psychoanalytic process we are talking about gradual, progressive steps in such a direction).

Indeed, neural network theorists have wrestled for a long time with the problem of how not to forget established facts and connections when you learn new facts and connections. How, for example, as children do we learn the faces of our schoolmates without forgetting the faces of our parents? This has been described as the problem of catastrophic forgetting (McCloskey and Cohen, 1989) or the stability-plasticity dilemma (Carpenter and Grossberg, 1987).

One of the goals of psychoanalysis, particularly in our modern version, is to promote mental flexibility and openness to experience. Mental flexibility depends fundamentally on the plasticity of neural connections, which was discovered in the 1960s by the Nobel neurophysiologist Eric Kandel (see in particular Kandel and Tauc, 1965). Indeed, Kandel (1999), a native of Vienna with a long-standing interest in Freud’s work, noted the contribution of neural plasticity to several of the key concepts of psychoanalysis.

Flexibility is essential to overcoming the either-or dichotomies of daily life. One of the most important of these dichotomies is self vs. others, otherwise expressed as self-interest vs. empathy. Now we ask what is the neural basis for our constant dance between concern for others’ wellbeing and our own wellbeing. We also ask how psychoanalysis can help us perform this dance better. Our inquiry will tie into the psychoanalytic concepts of narcissistic libido and object libido.

### Balancing self-interest and empathy in neural networks

To understand the biological roots of self-interest and empathy, we need an excursion into human evolution. The behavioral neuroscientist Paul MacLean (1990) developed his idea of the *triune brain*, the three parts of the brain that he labeled reptilian, old mammalian, and new mammalian. MacLean emphasized that in evolution the human mind/brain retained a great many processes from other animals but built on top of those processes. Yet he noted some fundamental changes as one ascends the evolutionary ladder.

Reptiles share with mammals many of the deeper brain structures. These structures enable reptiles to engage in many of the common behavior patterns also found in mammals, including humans. Lizards, for example, establish and defend territory; show domination and surrender; forage, hunt, and hoard food; form social groups and social hierarchies; greet and groom one another; court, mate, and breed (Paul MacLean, 1990).

Yet the lesser development of other brain areas means that reptiles, with some exceptions such as crocodiles, are less likely than mammals to engage in parental care. While reptiles typically spend efforts to tend and hatch their eggs before the birth of their young, many species of reptiles leave their young largely on their own after birth.

Parental care is the evolutionarily oldest form of concern for others. Caring parenting and other forms of bonding are the product of brain changes from reptiles to mammals. Specifically, mammals are more developed than reptiles in two brain areas called the thalamus and the cingulate cortex which are part of the neural pathways for expression of emotions.

The interdisciplinary social scientist Jerry Cory built on the triune brain idea to develop a theory of the dance in our brains between self-interest and empathy (Cory, 1999, 2004). Broadly, Cory located self-interest in MacLean’s reptilian complex—parts of the brain stem and basal ganglia. He said empathy was an offshoot of parental care, which he located in the old mammalian complex—parts of the limbic system. In his schema, both self-interest and empathy need to be attended to if they have been neglected too long. If self-interest is neglected, a person feels like life is unfair to them and they are sacrificing their wellbeing for others. If empathy is neglected, a person can degenerate into a state that is harmful for social interactions.

**FIGURE 2:**
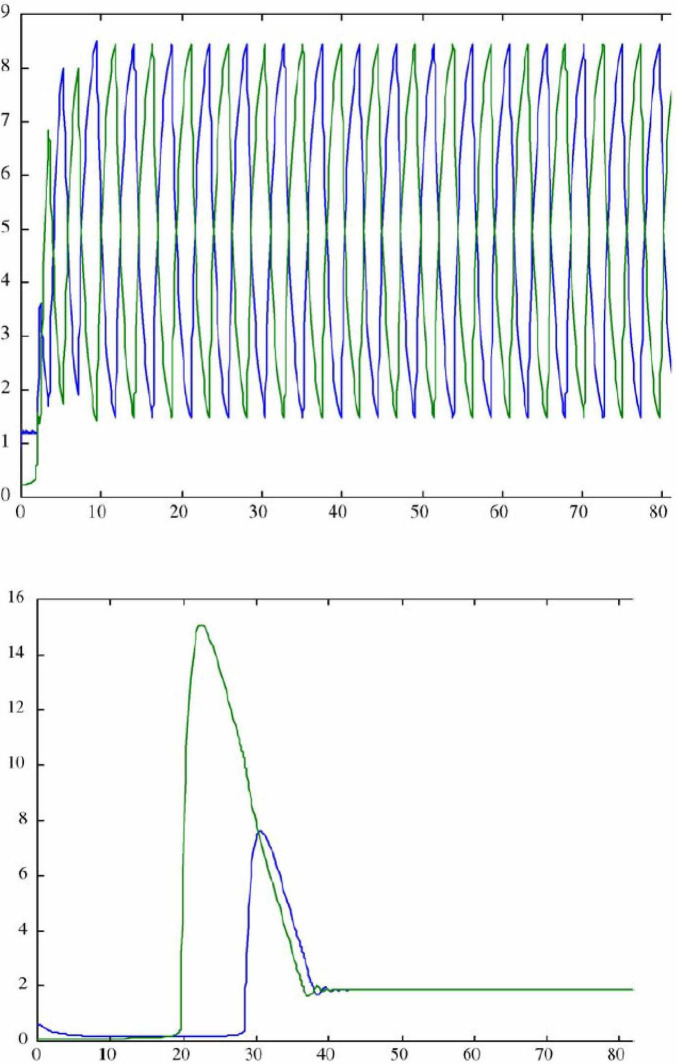
Top graph: dynamics of neural network with competing bias toward empathy and toward self-interest, without the stabilizing influence of prefrontal cortex. Bottom graph: dynamics of the same neural network with prefrontal cortex added (from Levine and Jani, 2002).

The self-interest/empathy dance has been modeled in a neural network (Levine and Jani, 2002; Levine, 2006, 2025; Levine and Kota, 2025). To a network similar to the “needs” network of Figure 1 some signals were added that represent biases in favor of either self-interest or empathy, with the bias shifting whenever the level of either self-interest or empathy gets too low. These signals are assumed to represent influences of the reptilian and old mammalian complexes, respectively. The top graph in Figure 2 shows the dynamics of this network over time: note that it moves continually back and forth between self-interest and empathy without reaching a balanced or equilibrium state. The network was then modified to include another area that is identified with a part of the brain’s frontal lobes (prefrontal cortex). The bottom graph of Figure 2 shows that with the “frontal lobes” included, the network reached a balanced state representing a healthy balance between empathy and self-interest, where a person has good social interactions but has a good relationship with their self.

Psychoanalysis aims to achieve this healthy balance. This includes a healthy ego, or sense of self, that interacts with other people without yielding to the super-ego that is dependent or submissive toward others.

### Healthy ego vs. punitive superego

Freud divided each human individuality into the well-known categories of id, ego, and superego. Roughly speaking, the id is the source of psychic energy that is present from birth; it is the more remote source of the Life Trieb and the locus of human Unconsciousness. The ego develops from the id, and from its interaction with the ego ideal and the superego, which function in different ways to allow the id contents to be expressed in the real world. The superego is the latest component to be fully develop in the child and it evolves from a very important psychic instance named “ego ideal” (that starts very early in the infant and lasts as long as the superego in human psychism) and provides ideals, ethical/moral standards and guidelines for making judgments.

It is very difficult to describe a single definition of “ego ideal”, not only because it is often connected with the concept of ‘superego’, but also because there is a variety of conceptions in the literature. For the objective of this paper, “ego ideal” is aligned with definitions that consider it as a “personality instance resulting from the convergence of the narcissism and the identification with the parents, its substitutes and with collective ideals” (Laplanche and Pontalis, 2001, p. 222). It is a reference for the ego to appreciate its accomplishments. We consider its psychic function very relevant, especially in the struggle between Death and Life Triebe.

Ego, id, and superego (and also ego ideal) represent the actions of brain networks that are always present, yet the balance between them varies between individuals. To understand their possible network dynamics, we can start with ego and id. Id would seem to be closely tied to emotional regions of the brain, specifically the hypothalamus and the amygdala. The development of the ego—which also involves the appearance of consciousness—would seem to be parallel that of the orbitofrontal cortex (OFC), which is the region of the brain’s cerebral cortex that connects directly with emotional regions, particularly amygdala (Schore, 1994). The OFC like the rest of the frontal lobes develops slower than the amygdala, and Schore asserts that parents of a very young child play the role of “substitute OFC” for the child.

Now what does this say about the “dance” between emotion and reason in the brain? The link between amygdala and OFC is two-way. There are more connections from amygdala to OFC than the reverse, but the reverse connections play an important role in the cognitive control of emotion, as when we consciously try to feel less bad about an unpleasant event (e.g., Ochsner and Gross, 2002). Probably, at those moments of emotional control, the superego is also active. Connections from OFC to amygdala we would expect to be both excitatory and inhibitory, as throughout life we consciously encourage healthy emotional responses while attempting to suppress unhealthy emotional responses. Marsha Linehan, a psychotherapist specializing in borderline personality disorders, discusses the need to integrate of our “rational self” and our “emotional self” into our “wise self” (Linehan, 1993).

The plasticity of the brain (Kandel, 1999; Kandel and Tauc, 1965) means that the connectivity between these two regions is heavily influenced by life experience, including the early influence of parents, culture, and religious upbringing. If this life experience is restrictive, giving messages of conditional positive regard (Rogers, 1951), we would expect to see an imbalance toward inhibition of the emotional id, which would mean an excess of inhibitory connections from OFC to amygdala. In other words, the overly punitive superego could be tied to a form of ultra-rationalism, or to an emotional fear of one’s own emotions. In the brain, some of the other areas of cortex involved in complex reasoning, such as the dorsolateral prefrontal cortex and the posterior parietal cortex, could play a role as well.

If the superego can be kept in check, as is one of the goals of psychoanalysis, the individual develops a strong sense of self and their own values. But it’s often necessary that the ego ideal—which has a softer and more amorous control over the ego than the superego –also have influence in this dynamics. People who have a good balance between the superego and the ego ideal are not overly dependent on the approval of others for their actions. Yet clearly, healthy relationships with and considerations for the interests of other people are an essential part of healthy adjustment. The research psychiatrist Robert Cloninger mapped out the likely growth of a healthy individual over their lifetime, marked by trade-offs between three dimensions of character that he called self-directedness, cooperativeness, and self-transcendence (Cloninger, 1999). As for brain mechanisms, Cory (1999, 2004) related self-directedness to MacLean’s reptilian complex (the brain stem and basal ganglia) and cooperativeness to MacLean’s old mammalian complex (the limbic system).

The trade-off between self-directedness and cooperation provides a new way of examining the psychoanalytic concepts of narcissistic libido and object libido. These are main sources of psychic energy and among the principal axes to understand the new innovative trends of contemporary psychoanalysis.

### Narcissistic and object libido

Psychoanalysts define narcissistic libido as psychic energy directed at oneself. They define object libido as psychic energy directed at objects outside oneself, including other people. These two libidos are present in all of us, but can take on healthy or unhealthy forms depending on the balance between id, ego, and superego and ego ideal

The term “narcissistic libido” has unpleasant associations to many of us because narcissism is the term used for excessive interest and preoccupation with oneself to the exclusion of consideration for other people. Narcissism is commonly grouped in the “Dark Triad” with the two other disorders of psychopathy and Machiavellianism (Doerfler et al., 2021). Yet the maladaptive form of narcissism typically coexists with a relatively weak sense of self, so is more likely if the frontal lobe dynamics of the ego are not well organized. A healthy, adaptive narcissistic libido, based in a strong prefrontally mediated sense of self, expresses legitimate self-esteem in order to interact well with other people.

Object libido could also be either unhealthy or healthy. If the superego is overactive as compared to the ego, object libido can be obsessive or reactive toward people or other objects. It can be unequal, typically subservient to other people’s demands or expectation. Yet in the presence of a strong ego, object libido, that, in this case, is more influenced by ego ideal than by superego, so can promote cooperative or empathic identification with others and their interests. In a person with a weak sense of self, narcissistic and object libido are likely to be in opposition, perhaps as competing attractors. Yet with a strong, mature sense of self as psychoanalysis tries to promote, the two libidos are likely to work together for the benefit of the person and their social interactions.

### Transference

Another key psychoanalytic concept to be understood in neural terms is transference. Transference is when feelings related to previous events in a person’s life are transferred to another person who is present. Usually this means the analysand transferring feelings related to their parents, partners, or other significant others, to the analyst. When the analyst transfers feelings from their past to the analysand, that is called countertransference.

For transference to work effectively, a high degree of trust between the analyst and analysand is essential. The hormone oxytocin, and its brain receptors in the nucleus accumbens of the basal ganglia, have been shown over decades to be essential for interpersonal trust. Oxytocin mediates pair bonding in rodents (Insel, 1992) and intranasal administration of oxytocin increases the ability of men playing an investment game to trust their partners (Kosfeld et al., 2005).

Trust is considered an emotional connection, which can of course get quite strong in the course of successful psychoanalysis. Yet years of experience indicate that transference is more effective when there is also a good intellectual relationship between the analyst and analysand. This is part of the “dance” between emotion and reason: emotional empathy, the ability to feel with what emotions another person is undergoing, needs to be supplemented by cognitive empathy, the ability to take the perspective of another person and understand where they are coming from, even if that other person is dissimilar from oneself. Different areas of the brain are involved in cognitive vs. emotional empathy, with one older area of the cerebral cortex called the insula possibly involved in both (Levine, 2021, p. 30–32). Emotional empathy without cognitive empathy carries with it the danger of putting people into in-groups and out-groups, leading to emotional contagion with the in-group and unconcern, or even hostility, toward the out-group (De Dreu, 2012). We speculate that perspective taking in the brain also involves the furthest forward part of the prefrontal cortex, also called the frontopolar cortex, which processes the most abstract concepts (Christoff and Gabrieli, 2000). Abstraction enables people to see similarities with individuals who are superficially dissimilar, as on ethnicity, nationality, profession, or life experiences.

The value of transference is an example showing that psychoanalysts tend to believe in redirecting rather than suppressing positive energy when it is not directed toward the most appropriate object. Another example of psychoanalytic redirection of energy is sublimation.

### Sublimation

Freud defined sublimation, in his first attempts to do it, as the redirection of socially unacceptable impulses, particularly sexual or aggressive impulses, toward socially acceptable ends. But this definition is very simple and did not satisfy its creator. Sublimation is one of the concepts that were difficult for Freud himself to theorize; it is well known that he destroyed the unfinished work he had written on it. In contemporary psychoanalysis, as in our research group, we look for more extensive re-readings of sublimation in Freud’s oeuvre, as well as a possible new psychic mechanism that can emerge from classical ‘sublimation with a greater potential of helping the individual to turn the Death Trieb into Life Trieb, perhaps the Holy Grail of our discipline research.

To understand sublimation at the neural network level, we first need to understand the structure of rules for behavior. Each one of us has internalized rules for what behaviors we should perform: based on analogies from an Escher painting and from immunology, Levine (2005) called those rules Angels. Each of us also has internalized rules for behaviors we should not perform: Levine (2005) called those rules Devils. In the same article, large classes of related Angels and Devils are called Censors. An example of a Censor would be that sexual intercourse is acceptable with one’s primary partner but with nobody else. Healthy as well as neurotic individuals have Angels, Devils, and Censors but different ones. The goal of psychoanalysis is to move the individual toward Censors that are more enhancing of the Life Trieb. But perhaps the Censors can perform various different roles than the ones society already admits and open a way to the new psychic mechanisms that we are looking for, besides sublimation.

**FIGURE 3:**
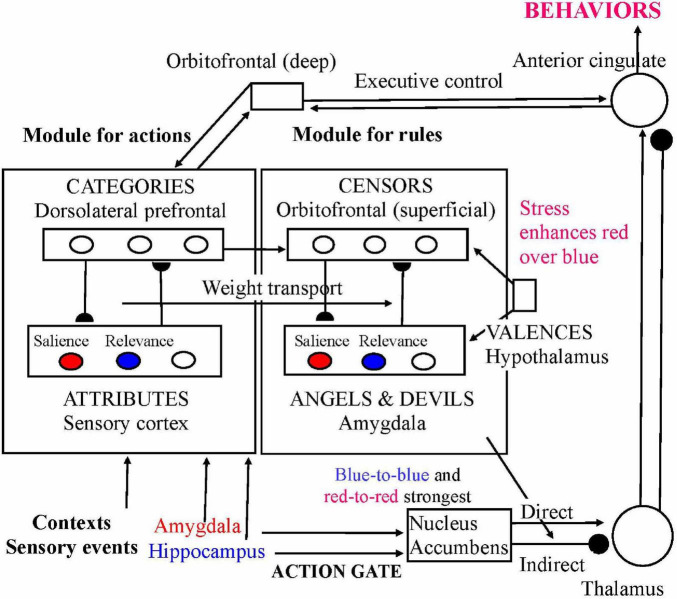
Possible (simplified) neural circuit connecting angels, devils, and censors. (i) The action module connects attributes and categories of possible actions, with the categories coding patterns of attribute values which are modifiable by bottom-up and top-down connections. Two of the attributes are emotional salience and task relevance. Relative weight of attributes can change with context: amygdalar activation (increased by severe or persistent stress) selectively enhances the weight of salience, whereas hippocampal activation (decreased by stress) enhances the weight of relevance. Blue denotes connections that are weakened by stress; red denotes connections that are enhanced by stress. (ii) The categorization module ‘lifts’ to another module connecting angels and devils (i.e., simple behavior rules) with censors (categories of behavior rules). Part of the amygdala, through conjunction of attribute signals from the cortex and affective valences (+ or –) from the hypothalamus, encodes attachment of positive or negative valence to attributes of behaviors. Part of orbitofrontal cortex likewise encodes attachment of positive or negative valence to categories of behaviors. (iii) The striatum, influenced both by emotional salience and task relevance, selectively activates (via the direct pathway to thalamus) or inhibits (via the indirect pathway to thalamus) motor implementations of specific actions at the anterior cingulate. (iv) The anterior cingulate and deeper layers of the orbital prefrontal cortex exert top-down executive control on both modules. This executive system responds to context and thus tends to enhance activities of task relevant representations. Reprinted by permission from Levine (2005).

Figure 3 depicts Levine (2005) tentative theory of the involvement of brain regions in the structure of Angels, Devils, and Censors. The complex interplay depicted in that figure between sensory cortex, prefrontal cortex, amygdala, hippocampus, hypothalamus, and basal ganglia serves to implement the feedback between specific and general behavioral rules. Activation of a general Censor tends to activate Angels and Devils related to that Censor, and vice versa.

A Censor can be broad enough to relate to a wide range of Angels. For example, sublimation of sexual impulses is related to the Censor that favors creative expression, which is what psychoanalysts call libidinal energy. Creative expression can manifest through sexuality (the urge to create offspring) or else through creativity in other domains such as the arts, science, and entrepreneurship. We propose that there is mutual inhibition between representation of different Angels within the same Censor. Such lateral inhibition between representations at the same brain level is common in computational models of neural processes, starting with sensory processes such as vision (Levine, 2019, Chap. 4). Among other brain regions, that type of mutual inhibition is found in the amygdala, which is the location for Angels in the network of Figure 3, where larger neurons inhibit one another indirectly via smaller interneurons (John et al., 2016, 2022).

Sublimation, in our theory, works via selective disinhibition of Angels. For example, say, there is mutual inhibition between the Angel favoring sexual expression and the Angel favoring artistic expression, both part of the Censor favoring creativity. If the sexual Angel is inhibited, that releases the artistic Angel from inhibition and allows it to be activated.

New body/mind dynamics can be proposed if psychoanalysis and neurosciences can join efforts to advance our knowledge in the new psychic mechanism we are looking for further than sublimation and a simplistic explanation of good vs. bad, moral vs. immoral, death vs. life Triebe. This interdisciplinary close collaboration can be an important element to help human beings to achieve the best that reason and emotion can provide to humanity: the continuous exercise and development of human capacity to experience wisdom and compassion toward themselves and the others.

### Toward an overarching theory

The multiplicity of models in Figures 1–3 for different aspects of the psychoanalytic process indicates involvement of the entire brain, including regions of the brain primarily involved in instincts, emotions, and thoughts (Levine, 2017; Paul MacLean, 1990). This leads us to seek a unified model of the psychoanalytic process that encompasses all of its different aspects, including switches between behavioral attractors, the Life and Death Triebe, narcissistic and object libido, ego ideal, transference, and sublimation.

**FIGURE 4:**
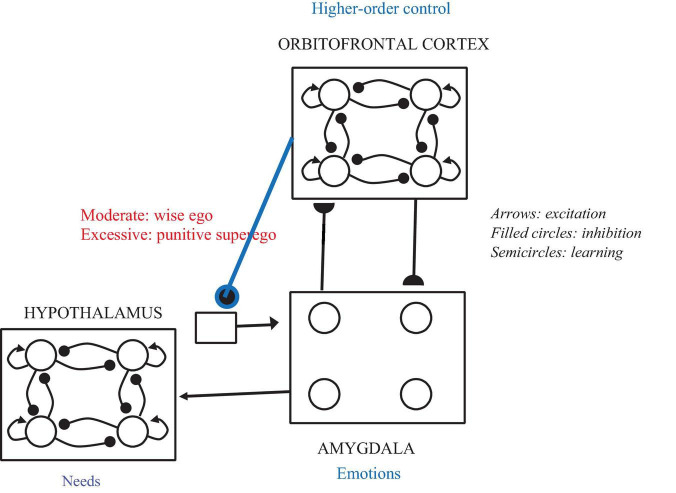
Diagram of the part of the brain-mind network on which the wise ego can operate. The amygdala and orbitofrontal cortex are connected via a two-layer network with modifiable synapses in both directions, in the manner of the adaptive resonance (ART) network of Carpenter and Grossberg (1987). The orbitofrontal cortex exerts higher-order controls via tonic inhibition (a feature of ART) on the emotional responses represented in the amygdala. If that inhibition is excessively strong, emotional needs tend to be suppressed by a punitive superego. If that inhibition is moderate, unhealthy emotions will be suppressed and healthy emotions enhanced, in the manner of the wise ego. The more activation remains at the amygdala after tonic inhibition, the more different needs can be satisfied by activation of the appropriate node in the hypothalamus.

All of these phenomena could be seen as arising out of a person’s quest to live by the dictates of the “wise self” of Linehan (1993) which encompasses and transcends both the rational and emotional selves. To bring it in line with psychoanalytic concepts, we rename it the wise ego (see Figure 4).

In neural terms, the wise ego is based on connections from the frontal lobes to the amygdala that are balanced between excitation and inhibition. In fact, one of the goals of psychoanalysis is to heal the connections between emotion and reason when needed (cf. Levine, 2021). Figure 4 shows in neural network terms how to synthesize, and thereby transcend, reason and emotion.

As for switches between behavioral attractors, recall that in the needs network of Figure 1, a sufficiently but not excessively high level of norepinephrine (initiative) tends to bias the network toward attractors that satisfy a greater number of needs. Lower levels of initiative tend to bias the network toward attractors that satisfy some needs at the expense of others. In particular, high levels of initiative, bolstered by analysis, make it more likely that the analysand will achieve a state that meets both the rational need for competence and knowledge of the environment and the emotional need for connectedness with others. As Levine (2021, p. 111) stated: “we need emotion to feel the importance of doing something and we need reason to carry it out effectively.” This is related to the ego ideal which works with the id rather than the superego which punishes the id. It is also related to the formulation of Cloninger (1999) who traced healthy human development to reaching high levels of three character dimensions: self-directedness, cooperativeness, and self-transcendence. In Cloninger’s system there is a “cube” where each of the eight corners represent an array of presences or absences of these dimensions, and these corners are attractors for the non-linear dynamical system representing personality. The wise ego is akin to Cloninger’s “creative” state, the corner of his cube where all three dimensions are high.

As for the Life and Death Triebe, the wise ego employs both Triebe for growth under different circumstances. We have discussed the connection of the Death Trieb with taking risks, which we all need to do in order to have a meaningful life. There can be risks of being unsuccessful in reaching a goal, or of being regarded badly by others, or in some cases even of death itself. So the Death Trieb is necessary to accept the possibility of defeat or of death. The Death Trieb helps us overcome the obsession with mere survival in favor of a desire to live in a way that makes a difference to others, including others who are not yet born. Eisler and Levine (2002) discussed three different responses to stressful situations: fight-or-flight, dissociation, and tend-and-befriend, all of which we need to be available at different times. And just as tend-and-befriend is closely tied to the Life Trieb, fight-or-flight—the need to defend what we value—is tied to the Death Trieb. (Dissociation is a third mode, which we need sometimes to rest and restore energy.) Meaningful life again requires that we strive to meet our needs both for competence in the world and for relatedness to others.

As for narcissistic and object libido, the wise ego means that prefrontal influences on the amygdala are balanced between excitation and inhibition. Again this means that Cloninger’s variables of self-directedness and cooperativeness are both high, and the ego ideal is stronger than the potentially punitive superego. This means that the narcissistic libido, the energy directed to oneself, is based in positive self-esteem and not a craving for attention. Conversely, the object libido, the energy directed at other people and the environment, is oriented toward wise interactions and not exploitation or overdependence.

As for transference, the ego ideal fosters the harmonious relationship between reason and emotion which facilitates both intellectual and emotional connection between analysand and analyst. In this connection, compassion and caring are combined with nuanced understanding of the analysand’s situations, including honest appraisal of difficulties in the analysand’s life and behavior.

As for sublimation, the wise ego does not suppress emotional desires that are not optimally directed. It sees these desires as part of the narcissistic and object libidos which energize the analysand and aims to find behavioral outlets that can redirect those desires toward something similar to, yet different from, their original object.

## Conclusion

Galatzer-Levy (2017) notes that psychoanalysis, and other forms of psychotherapy, benefit from incorporating insights from the mathematics of non-linear dynamical systems. In linear systems, small changes in outside inputs lead to small changes in behavior, whereas in non-linear systems, small changes in outside inputs can often lead to abrupt changes in behavior. Hence, small changes in interactions with significant others, including the analyst, can move an analysand back and forth between an unhealthy and a healthy pattern (attractor) of behavior, thought, or emotion.

In fact, healthy adjustment includes storing multiple attractors in one’s behavioral repertoire and switching between attractors as outside circumstances change. An apt analogy comes from martial arts such as Japanese karate, in which the practitioner’s body is normally in a calm, peaceful posture but then switches to a fighting posture when confronted with an adversary.

Edalat and Lin (2014) developed a neural network model of the process of psychotherapy. Their model is based on the theoretical work of Levine (2005, 2009) and Aleksandrowicz and Levine (2005), including interactions between amygdala, OFC, DLPFC, and ACC and a competitive network representing needs of the organism. Edalat and Lin simulated the process of changing from heuristic, emotionally based decisions to mindful, deliberate decisions. They note that their model is particularly applicable to cognitive behavioral therapy (CBT) mindfulness based CBT, and psychodynamic psychotherapy.

Our model based on Figure 4 has much in common structurally with Edalat and Lin’s but is different in some important respects. First of all, psychoanalysis differs from mindfulness based therapies in that it goes further in exploring unconscious motives, including those derived from the analysand’s early childhood. Thus at its best, psychoanalysis engages a larger part of the brain, including all three evolutionary layers described by Paul MacLean (1990) and their interactions. Also, more than Edalat and Lin’s, our approach transcends the dichotomy between reason and emotion that is harmful both to the individual and society, in favor of the “wise ego” that integrates reason and emotion, deliberation and intuition.

Future developments of the model will incorporate the properties of different presumed needs in the network of Figure 4, with different influences from amygdala and orbitofrontal cortex. This can lead to simulations of different stages, including ups and downs, in the psychoanalytic process, in which the “wise ego” integration of emotion and reason is sometimes successful and sometimes problematic. Some previous models in other domains suggest fuzzy similarity structures between possible states (Versaci et al., 2025). Yet the range of possible attractors and intermediate non-equilibrium states in our model is practically limitless and likely to capture the variety of psychological states in individuals undergoing psychoanalysis.

## Data Availability

The original contributions presented in this study are included in this article/supplementary material, further inquiries can be directed to the corresponding author.

## Footnotes

1 We use the German word “Trieb” that Freud used because it comes closer to our intended meaning than the closest English equivalent, which is “drive.”

2 The great difficulty of citing, in a text of original reinterpretation of Freudian concepts, lies on the fact that they were re-presented by Freud countless times throughout his work, rarely in a way that completely annulled his previous definition. In this article, based on our rereading of the Freudian Work, without neglecting essential aspects of his meta-psychological texts, we consider that Life Trieb can be understood as the energy that impels us to live and face all possible difficulties to keep living, and the Death Trieb can be defined as an expression of the refusal of this vital movement that can be negative if it stops it for long time—in a regressive way—or positive if it represents a moment of retreat for recovery, or even improvement, from excessive or equivocal psychic investments.
